# Synaptic proteasome is inhibited in Alzheimer’s disease models and associates with memory impairment in mice

**DOI:** 10.1002/alz.088220

**Published:** 2025-01-03

**Authors:** Felipe C Ribeiro, Danielle Cozachenco Ferreira, Luana Heimfarth, Juliana Fortuna, Guilherme B. de Freitas, Jorge M Souza, Soniza Alves‐Leon, Renata Elaine Paraizo Leite, Claudia Kimie Suemoto, Lea T. Grinberg, Fernanda G De Felice, Mychael V Lourenco, Sergio T Ferreira

**Affiliations:** ^1^ Federal University of Rio de Janeiro, Rio de Janeiro, Rio de Janeiro Brazil; ^2^ Federal University of Rio de Janeiro, Rio de Janeiro Brazil; ^3^ Queen’s University, Kingston, ON Canada; ^4^ Clementino Fraga Filho University Hospital, Rio de Janeiro Brazil; ^5^ University of São Paulo Medical School, São Paulo, São Paulo Brazil; ^6^ Weill Institute for Neurosciences, University of California San Francisco, San Francisco, CA USA; ^7^ Institute of Medical Biochemistry Leopoldo de Meis, Federal University of Rio de Janeiro, Rio De Janeiro, Rio de Janeiro Brazil; ^8^ Federal University of Rio de Janeiro, Rio de Janeiro, RJ Brazil

## Abstract

**Background:**

The proteasome plays key roles in synaptic plasticity and memory by regulating protein turnover, quality control, and elimination of oxidized/misfolded proteins. Here, we investigate proteasome function and localization at synapses in Alzheimer’s disease (AD) post‐mortem brain tissue and in experimental models.

**Method:**

We used primary hippocampal cultures, amyloid‐β oligomers (AβO)‐injected or transgenic animal models, and human brain tissue to determine brain proteasome function and subcellular localization. We further induced proteasome inhibition in vitro and in vivo to determine AD‐like phenotypes and memory in mice.

**Result:**

We found a marked increase in ubiquitinylated proteins in post‐mortem AD hippocampi compared to controls. Using several experimental models, we show that AβO inhibit synaptic proteasome activity and trigger a reduction in synaptic proteasome content. We further show proteasome inhibition specifically in hippocampal synaptic fractions derived from APPswePS1ΔE9 mice. Reduced synaptic proteasome activity instigated by AβOs is corrected by treatment with rolipram, a phosphodiesterase‐4 inhibitor, in mice. Results further show that dynein inhibition blocks AβO‐induced reduction in dendritic proteasome content in hippocampal neurons. Finally, proteasome inhibition induces AD‐like pathological features, including reactive oxygen species and dendritic spine loss in hippocampal neurons, inhibition of hippocampal mRNA translation, and memory impairment in mice.

**Conclusion:**

Results suggest that proteasome inhibition may contribute to synaptic and memory deficits in AD.

